# Emergency Medical Service Response Time for Road Traffic Accidents in the Kingdom of Saudi Arabia: Analysis of National Data (2016–2020)

**DOI:** 10.3390/ijerph20053875

**Published:** 2023-02-22

**Authors:** Thamer Alslamah, Yousef Mohammad Alsofayan, Mahmudul Hassan Al Imam, Monerah Abdullah Almazroa, Adil Abalkhail, Ibrahim Alasqah, Ilias Mahmud

**Affiliations:** 1 Department of Public Health, College of Public Health and Health Informatics, Qassim University, Al Bukairiyah 52741, Saudi Arabia; 2 Executive Administration of Medical Affairs, Saudi Red Crescent Authority, Riyadh 11129, Saudi Arabia; 3 School of Health, Medical, and Applied Sciences, Central Queensland University, Rockhampton, QLD 4701, Australia; 4 Central Queensland Public Health Unit, Central Queensland Hospital and Health Service, Rockhampton, QLD 4701, Australia; 5 Emergency Medical Registry Department, Saudi Red Crescent Authority, Riyadh 11129, Saudi Arabia

**Keywords:** road traffic accidents, accidents management, accidents response, Saudi Arabia, Red Crescent Authority, Red Crescent, response time

## Abstract

Despite preventive measures and initiatives, road traffic accidents are on the rise in the Kingdom of Saudi Arabia. This study aimed to investigate the emergency medical service unit’s response to RTA by socio-demographic and accident-related variables in the Kingdom of Saudi Arabia. This retrospective survey included Saudi Red Crescent Authority data on road traffic accidents between 2016 and 2020. As part of the study, information on sociodemographic characteristics (e.g., age, sex, and nationality), accident-related data (type and place of the accident), and response time to road traffic accidents were extracted. Our study included 95,372 cases of road traffic accidents recorded by the Saudi Red Crescent Authority in the Kingdom of Saudi Arabia between 2016 and 2020. Descriptive analyses were performed to explore the emergency medical service unit’s response time to road traffic accidents, and linear regression analyses were performed to investigate the predictors of response time. Most of the road traffic accident cases were among males (59.1%), and the age group of 25–34 years accounted for about a quarter (24.3%), while the mean age of the road traffic accident cases was 30.13 (±12.86) years. Among the regions, the capital city of Riyadh experienced the highest proportion of road traffic accidents (25.3%). In most road traffic accidents, the mission acceptance time was excellent (0–60 s; 93.7%), movement duration was excellent (<120 s; 91.1%), reaching site duration was excellent (<12 min; 57.9%), treatment start time was excellent (<120 s; 76.4%), duration at the scene was poor (>15 min; 40.8%), reaching hospital duration was good (30–60 min; 52.7%), and in-hospital duration was poor (>15 min; 44.1%). Regions, places and types of accidents, age, gender, and nationality of victims were significantly associated with different parameters of response time. Excellent response time was observed in most of the parameters except the duration at the scene, reaching hospital duration, and in-hospital duration. Apart from the initiatives to prevent road traffic accidents, policymakers should focus on strategies to improve accident response time to save lives.

## 1. Introduction

Road traffic accidents (RTA) are considered a global public health concern due to their serious impact on health and the economy. The number of deaths due to RTA is on the rise, from 1.2 million in 2018 to 1.4 million in 2020 globally [1]. RTA is the eighth cause of mortality worldwide. Considering its devastating impacts, the United Nations General Assembly has given a target of halving the total deaths and injuries from RTA by 2030 [2]. According to the World Health Organization (WHO), 3% of the total gross domestic product (GDP) is spent on managing RTAs in most countries [2].

RTA is the second leading cause of death and primary cause of premature death in the Kingdom of Saudi Arabia (KSA). The Global Status Report on Road Safety 2018 identified the KSA with the highest mortality rate from RTAs in high-income countries (HICs) [3]. In 2016, an estimated 28.8 per 100,000 population (i.e., 9031) deaths from RTAs were reported in the KSA; which was much higher compared to the average mortality of 9.2 per 100,000 reported in HICs [3]. According to the Ministry of Health of the KSA, 28,123 people were injured due to RTAs in 2020 [4]. RTA is one of the leading causes of acquired disabling conditions such as spinal cord injury; 85% of traumatic spinal cord injuries are caused by RTAs in the KSA [5]. Furthermore, the economic impact of RTAs is extremely high; it costs USD 3.75 billion, approximately 2% of the national GDP in 2016–17 [3]. However, the actual economic cost is expected to be much higher as it should include the cost of healthcare and rehabilitation as well as the productivity loss due to premature deaths or disability [6].

The WHO has identified five Road Safety Pillars, including (a) road safety management or policy; (b) road infrastructure; (c) safe vehicles; (d) safe behavior of road users; and (e) post-crash care [7]. The response time for post-crash care can be defined as the time elapsed between notification of accidents and arrival on the scene, which is crucial to facilitate survival [8]. The early arrival of the emergency medical care team can ensure the stabilization of individuals with life-threatening injuries, timely triage, and hospitalization [9,10]. In contrast, delays in response time could increase the risk of death [8]. Byrne et al. [8] observed that a longer response time is associated with a higher fatality rate in the United States of America (USA). Sanchez et al. [11] observed that a 10 min reduction in the response time can reduce the likelihood of death by one-third. Assessment of response time has been found effective in identifying the gaps and scopes for improvement in the accident management systems in Abu Dhabi [12]. However, such evidence is lacking in the KSA.

Existing studies indicate that the accident management system and response time are substandard in the KSA. The average RTA response time to road traffic crashes is 14 min, which is almost double compared to the international standard (i.e., 8 min) [13]. Mansuri et al. [14] reported that post-crash care is often delayed, which contributes to the rise of deaths and disabilities in the KSA. Al-Zabidi et al. [13] also reported that the long response time is associated with the geographical region, crash time, number of injured people, type of injury, and weather conditions in the KSA. To date, no studies have reported the impact of socio-demographic characteristics on RTA response time. Therefore, this study examined retrospective records on RTAs by the Saudi Red Crescent Authority (SRCA) covering the 2016–2022 period to investigate the Emergency Medical Services (EMS) unit’s response time to RTAs and sociodemographic and accident-related predictors of these response times.

## 2. Methods

We analyzed retrospective national data on RTAs from 2016 to 2020, collected and stored by the SRCA. SRCA offers emergency medical services (EMS) for RTA cases in all administrative areas of the KSA. EMS service can be requested by dialling 997. All RTA cases attended by the EMS unit of the SRCA during the 2016–2020 period were included in this study. We removed duplicate cases after filtering using the unique case ID. The entries with incomplete information and missing variables were also excluded from the study.

Explanatory/independent variables included socio-demographic information of the RTA cases—age, sex, nationality, and region, and accident-related information—the type of the accident, and place of the accident. Our dependent variables included the EMS unit’s response time to RTA events. These response times included mission acceptance duration, movement duration, reach site duration, onsite treatment start duration, duration at the site, reach hospital duration, and in-hospital duration. Mission acceptance duration was defined as the time from the EMS unit receives a mission request to the time the EMS unit accepts it. This variable was classified into 0–60 s (Excellent), 61–120 s (Good), and >120 s (Poor). Movement duration was defined as the time from the EMS unit receives a mission request to the time the EMS unit starts the movement to the incident site. This variable was classified into: <120 s (Excellent), 120–180 s (Good), and >180 s (Poor). Reach site duration was defined as the time from the EMS unit receives a mission request to the time the EMS unit reaches the incident site. This variable was classified into: <12 min (Excellent), 12–16 min (Good), and >16 min (Poor). Onsite treatment start duration was defined as the time from the EMS unit reaching the site of the incident to the time the EMS unit starts treatment at the incident site. This variable was classified into: <120 s (Excellent), 120–180 s (Good), and >180 s (Poor). Duration at the site was defined as the time from the EMS unit reaching the site of the incident to the movement of the EMS unit from the site of the incident. This variable was classified into: <10 min (Excellent), 10–15 min (Good), and >15 min (Poor). Reach hospital duration was defined as the time taken by the EMS unit to reach a hospital with an accident victim after they receive a mission request. This variable was classified into: <30 min (Excellent), 30–60 min (Good), and >60 min (Poor). In-hospital duration was defined as the time from the EMS unit reaches the hospital to the EMS unit leaves the hospital. This variable was classified into: <10 min (Excellent), 10–15 min (Good), and >15 min (Poor).

We did descriptive analyses using the Statistical Package for the Social Sciences (SPSS) version 20, while linear regression analyses were performed using the STATA version 8 SE. We did descriptive analyses of socio-demographic and accident-related variables and the EMS unit’s response times to RTA events. For descriptive analyses, we reported frequencies and percentages. For linear regression analyses, we reported unstandardized coefficients with a 95% confidence interval. A *p*-value of <0.05 was considered statistically significant.

## 3. Results

Our study included 95,372 cases of RTA recorded by the SRCA in the KSA between 2016 and 2020. Table 1 presents the characteristics of the RTA cases recorded during this period. We found that during this period, the highest number of accidents were recorded in Riyadh (25.3%) and Mecca (24.5%), and these two regions were followed by the western region (10.9%), Aseer (10%), Medina (6.4%), and Qassim (5.2%). The majority (68.6%) of accidents occurred inside cities. Among the RTA victims, 42.7% were Saudi nationals, about a fifth (19.3%) were non-Saudi nationals while nationality was unknown for 38.1% of cases. Most of the RTA cases were male (59.1%) while for 33.2% of cases, gender identity was not known. Among the RTA cases, about a quarter (24.3%) were aged between 25 and 34 years, and about a fifth (18.6%) were aged between 15 and 24 years. The mean age of the RTA cases was 30.13 (±12.86) years. We found that most of the RTA cases were collisions (76%), which were followed by roll-over (15%) and run-over (9%).

In most RTAs, mission acceptance time was excellent (0–60 s; 93.7%), movement duration was excellent (<120 s; 91.1%), reaching site duration was excellent (<12 min; 57.9%), the treatment start time was excellent (<120 s; 76.4%), duration at the scene was poor (>15 min; 40.8%), reaching hospital duration was good (30–60 min; 52.7%), and in-hospital duration was poor (>15 min; 44.1%) (Table 2).

### 3.1. Predictors of SRCA’s Response Time to Road Traffic Accidents

#### 3.1.1. Mission Acceptance Duration

We saw a significant association between mission acceptance duration and region, place and type of accident, nationality, gender, and age of the victims (Table 3). Compared to the Riyadh region, in all the other regions, significantly less amount of time (12.8 s less in Hail–2.1 s less in the western region) was needed to accept a mission. Being a non-Saudi victim was associated with 0.6 s higher time than a Saudi national in accepting a mission by the SRA. While compared to males, being a female victim was associated with 0.9 s less time in accepting a mission by the SRA. Compared to children aged 0–14 years, individuals aged 15–24 years or 25–34 years needed 1.3 s less time in accepting a mission. However, no significant differences in time to accept a mission was seen between the accident victims aged 0–14 years and 35–44 years, and 45–54 years and >54 years. Run-over accident was associated with 1.7 s less time to accept a mission compared to collision accidents. While no significant differences in time to accept a mission are seen between collision and rollover type accident. Accidents outside a city were associated with 7 s higher in accepting a mission by the SRA compared to an accident that happened inside a city.

#### 3.1.2. Movement Duration

Regarding the duration of the start to move to an accident site, we saw a statistically significant association between the time to start a movement and region, type and place of accident, nationality, and gender of the accident victims. Compared to the Riyadh region in all the other regions but Mecca and Al-Baha, SRA needed significantly less amount of time (35.6 s less in Hail to 7.9 s less in the western region) to start a movement to an accident site. Mecca and Al-Baha needed a significantly higher amount of time in this regard than Riyadh. Being a non-Saudi accident victim was associated with 2 s higher time to start a move to the accident sites than Saudi victims. While being a female accident victim was associated with a 1.8 s less time than a male to start a move to the accident site by the SRA. Compared to collision-type accidents, run-over type of accidents were associated with 5.1 s less time on average, while rollover type of accidents was associated with 3.1 s more time on average. Compared to inside-city accidents, outside-city accidents were associated with 22.3 s higher time to start a movement to the accident site by the SRA.

#### 3.1.3. Reach Site Duration

Regarding reach site duration, compared to the Riyadh region in Hail, Qassim, Aser, Jazzan, Najran, Al-Jouf, and Northern border regions, the SRA took significantly less time (3.1 min in the Northern border to 0.3 min less in the Aser region to reach an accident site). However, Medina was associated with a 0.7-min higher time to reach the site than Riyadh. For non-Saudi national accident victims, on average, the SRA took a significantly higher time (0.6–0.9 min; *p* < 0.001) to reach the site compared to the Saudi nationals. Compared to the youngest age group (0–14 years) accident victims, for older age group accident victims, the SRA took significantly less time to reach the accident sites (1.7 min less for the 15–24-year age group to 0.6 min less for the 45–54-year age group). Compared to collision-type accident, on average, run over-type accident was associated with 3 min less (−3.3 to −2.8; *p* < 0.001) in reaching the accident site by the SRA while rollover-type of accidents was associated with 2.3 min more time (2.1–2.5; *p* < 0.001) to reach the site. Our results also suggest that for outside city accidents, on average, the SRA takes 4.9 min more time (4.8–5.1; *p* < 0.001) to reach an accident site than inside city accidents.

#### 3.1.4. Onsite Treatment Start Time

We saw statistically significant relationships between onsite treatment start time and region, gender, type of accident, and place of accident. However, no statistically significant relationship was seen between the onsite treatment start time and the age and nationality of the accident victims (Table 4). Compared to the Riyadh region, in the north border (73.5 s less), Hail (56 s less), Al-Jouf (55.4 s less), Al-Baha (28 s less), Qassim (26.6 s less), Najran (18.2 s less), Western region (9.1 s less) the SRA took significantly less time to start onsite treatment after reaching the site. However, in the Aser (β = 22.4; *p* < 0.001) and Mecca region (β = 22.4; *p* < 0.001) the SRA took significantly more time than the Riyadh region to start onsite treatment after reaching the accident site. The female gender was associated with a significantly higher amount of time (16.6 s more) to start onsite treatment than the male gender (β = 16.6; *p* < 0.001). Rollover-type accidents were associated with a significantly higher amount of time (18.8 min more) to start onsite treatment than collision-type accidents (β = 18.8; *p* < 0.001). Outside-city accidents took a significantly lower amount of time (28.8 s less) to start onsite treatment after reaching the site than inside-city accidents (β = 28.8; *p* < 0.001).

#### 3.1.5. Duration Spent at the Accident Site

We saw statistically significant relationships between the duration spent at the accident site by the SRA and the region, nationality, gender, age, type of accident, and place of accident (Table 4). Our results suggest that compared to the Riyadh region, in all other regions but Mecca and Al-Jouf, the SRA spent significantly less time at the accident site. Al-Jouf region was associated with higher time spent at the accident site compared to the Riyadh region (β = 10.6; *p* < 0.001), while no statistically significant difference was overserved between the Riyadh and Mecca regions. Non-Saudi nationals were associated with spending significantly higher time (0.4 min) at the accident site by the SRA than Saudi nationals (β = 0.4; *p* < 0.001). Accident victims being female was associated with spending a significantly higher amount of time (1.6 min) at the accident site by the SRA than male genders (β = 1.6; *p* < 0.001). Compared to 0–14-year age group accident victims, 15–24-year-old age groups victims were associated with less time spent at the accident site (β = −0.6; *p* < 0.05). However, no statistically significant differences were overserved between 0–14-year age group and other age groups. Compared to the collision-type accident, run-over-type accidents were associated with significantly less time (β = −1.5; *p* < 0.001) while rollover-type of accidents were associated with significantly higher time (β = 0.7; *p* < 0.001) spent at the accident site by the SRA. On average, outside-city accidents were associated with less time (2.2 min) spent at the accident site by the SRA than inside-city accidents (β = −2.2; *p* < 0.001).

#### 3.1.6. Reach Hospital Duration

Our results suggest statistically significant associations between the time taken by the SRA to take the accident victim to a hospital after it received the mission request and the region, nationality, gender, age, accident type, and place of accidents (Table 4). Compared to Riyadh, the northern border region (19.1 s less) had the significantly least time taken to reach a hospital followed by Al-Jouf (17.6 s less) and Hail (12.1 s less). Non-Saudi nationals and females required 1.8 s and 3.1 s higher time to reach a hospital, respectively, compared to Saudi nationals and males. In terms of age, younger age groups required significantly less time to reach a hospital; 15–24 year-age groups required 7.6 s less time, 25–34 year-age groups required 6.4 s less time, 35–44 year-age group required 5.9 s less time, 55–54 year-age group required 6.2 s less time, and 55 years and above age group required 5.1 s less time to reach a hospital. Individuals with run-over accidents required 9.5 s less time, while those who had rollover accidents needed 7.0 s more time to reach a hospital. Outside-city accidents needed 8.6 s higher time to reach a hospital compared to accidents that occurred inside a city.

#### 3.1.7. In-Hospital Duration

We overserved evidence of statistically significant associations between the time spent in hospitals with accident victims by the SRA and the region, nationality, gender, age, accident type, and place of accidents (Table 4). Compared to Riyadh, both the north border and Hail regions (14.0 s less) had the significantly least time taken in a hospital followed by Al-Jouf (13.1 s less) and Qassim (11.0 s less). Non-Saudi nationals needed 0.8 s less time in the hospital compared to Saudi nationals. Females required 1.1 s higher time in the hospital compared to males. Compared to the 0–14-year age group, the 15–24 year-age group required 1 s less time in the hospital while both 25–34 year and 55 years and above age groups required 1.5 s less time. Individuals who had run-over accidents required 0.1 s higher time while individuals who had rollover accidents required 0.9 s higher time in the hospital. Outside-city accidents needed 3.6 s less time in a hospital compared to accidents that occurred inside a city.

## 4. Discussion

This study evaluated the accident response time of SRCA by socio-demographic and accident-related variables in the KSA. We found that the capital city Riyadh accounted for over a quarter of all RTA cases in the KSA between 2016 and 2020, and the Mecca region accounted for another quarter. We found that most of the accidents happened inside cities (68.6%). Most of the RTAs were reported in the age group of 15–44 years, with the 25–34-year age group accounting for a quarter of the cases. Among the RTA victims, males constituted the majority (59.1%). Our data suggest that SRCA’s EMS unit’s response time to RTA events was excellent for most of the parameters including mission acceptance time (0–60 s), movement duration (<120 s), reaching site duration (<12 min), onsite treatment-start time (<120 s). While the duration at the scene was poor (>15 min), the reaching hospital duration was good (30–60 min) and the in-hospital duration was poor (>15 min) for most of the cases. In terms of the predictors of SRCA’s EMS unit’s response time to RTAs, the mission acceptance duration, the movement duration, RTA site reaching duration, onsite treatment start-time, duration spent at the RTA sites, hospital reaching duration, and in-hospital duration were significantly associated with regions, place, and type of accidents, age, gender, and nationality of the victims.

This study revealed that young people (15–34 years) are more prone to RTAs in the KSA. Evidence suggests that driving just for fun (joyriding) and irritability are the common key reasons for young drivers’ involvement in RTA [15]. Because of their lack of expertise and greater carelessness, young people are more likely to be involved in an accident than middle-aged adults. Male genders were more predisposed to RTAs in our study than their female counterpart. This finding is supported by a previous study reporting 92% of RTA victims are males in Najran, KSA [16]. The government of KSA changed its law and started issuing driving licenses to Saudi women in 2018 [17]. A recent national-level survey found that only 23% of the women who earlier used to be car-passengers started driving by themselves [18]. This finding indicates that men are the most common driver in the KSA, which makes them more prone to this.

This study observed significant relationships between RTA response time with socio-demographic variables such as gender and nationality. Surprisingly, the female gender required a significantly higher response time in the onsite treatment start time domain, duration at site domain, reach hospital domain, and in-hospital domain. It could be partially explained by the cultural sensitivity around handling a female victim by male rescuers in the conservative society of the KSA. Furthermore, non-Saudi nationals observed longer response times in all domains compared to Saudi nationals. The policymakers of the KSA should take immediate steps to put a halt to gender-based and nationality-based discrimination which will eventually save hundreds and thousands of lives.

Our study also found that accident characteristics such as accident type, place, and region are strongly associated with RTA response time. An earlier study from the KSA reported that geographical region, type of injury, and weather conditions are significantly associated with response and rescue time [13]. Like our findings, Al Kabbi et al. [12] examined predictors of accident response time in Abu Dhabi and concluded that accident response time is significantly related to temporal characteristics, accident type, and location of the accident. In the USA, accident severity was reported to be the strong predictor influencing RTA response time [19]. Meng and Weng argued that the location of the RTA is a strong predictor of RTA response time [20]. In our study, the mission acceptance duration, movement duration, RTA spots reaching duration, and hospital reaching duration from RTA sites were higher for RTAs that occurred outside of cities. RTAs that happened outside of cities required higher response time due to the distance between SRCA stations and the accident site. Thus, special attention should be given to reducing the response time to RTA occurring outside of cities. Decentralization of existing EMS services might help in improving RTA response time.

In the KSA, one person is killed and four others are injured every hour due to RTAs [21]. Al-Ghambi [22] evaluated RSCA’s ambulance rescue time, including response time, time at the scene, and travel time to the hospital, and reported that the average rescue time is 35.84 min. The author argued that the operational procedures of SRCA should be reviewed to improve existing rescue time, which would save more lives. Strong and effective collaboration between the Ministry of Health and the Ministry of Transport and Logistic Services and other relevant organizations (e.g., RSCA) is necessary to achieve the targets of the global Decade of Action for Road Safety.

This study used national-level data from the RSCA register on RTAs in the KSA, which is a methodological strength of this study. However, this study is associated with some limitations. The influence of administrative and operational factors associated with response time was not investigated in our study. This study did not consider the severity of accidents, which is a significant predictor of RTA response time [19]. Additionally, the response time parameters used in this study have not been validated yet. We strongly recommend future studies with validated tools/parameters and accident severity and administrative and operational data.

## 5. Conclusions

To the best of our knowledge, this is the first study of its kind to evaluate the accident response time of the SRCA by socio-demographic and accident-related variables in the KSA. Our study found that most of the RTAs were reported in the age group of 25–34 years, and males had more RTAs than females. The capital city of Riyadh experienced the highest proportion of the RTAs followed by Mecca and the western region. Regarding response time, most of the parameters have observed excellent duration except duration at the scene (Poor, >15 min), reaching hospital duration (Good, 30–60 min), and in-hospital duration (Poor, >15 min). Regions, places and types of accidents, age, gender, and nationality of victims were significantly associated with different parameters of response time. Significant higher response time for females and non-Saudis demand further in-depth evaluation of the issue. Apart from initiatives to prevent RTAs, policymakers should focus on strategies to improve accident response time to save lives.

## Figures and Tables

**Table 1:** Place of accidents and characteristics of the road traffic accident victims in the KSA.

Variables		Frequency	Percent
The region where the accident took place	Riyadh	24,133	25.3
	Mecca	23,336	24.5
	Western Region	10,438	10.9
	Aser	9547	10.0
	Medina	6076	6.4
	Qassim	4933	5.2
	Jazzan	4481	4.7
	Tabuk	3275	3.4
	Hail	2867	3.0
	Al-Baha	1981	2.1
	Najran	1586	1.7
	Al-Jouf	1577	1.7
	North border	1142	1.2
Place of accident	Inside City	65,406	68.6
	Outside city	29,966	31.4
Nationality of the accident victim	Saudi	40,695	42.7
	Non-Saudi	18,360	19.3
	Unknown	36,317	38.1
Gender of the accident victim	Male	56,376	59.1
	Female	7356	7.7
	Unknown	31,640	33.2
Age of the accident victim	0–14 years	3509	3.7
	15–24 years	17,701	18.6
	25–34 years	23,154	24.3
	35–44 years	10,461	11.0
	45–54 years	4484	4.7
	55 and above	3431	3.6
	Unknown	32,632	34.2

**Table 2:** SRCA’s response time to road traffic accidents in the KSA.

Response Time	Frequency	Percent
Mission acceptance duration		
Excellent (0–60 s)	89,409	93.7
Good (61–120 s)	4007	4.2
Poor (>120 s)	1956	2.1
Movement duration		
Excellent (<120 s)	86,854	91.1
Good (120–180 s)	5481	5.7
Poor (>180 s)	3037	3.2
Reach site duration		
Excellent (<12 min)	55,182	57.9
Good (12–16 min)	15,769	16.5
Poor (>16 min)	24,421	25.6
Treatment start time		
Excellent (<120 s)	72,888	76.4
Good (120–180 s)	4481	4.7
Poor (>180 s)	17,993	18.9
Duration at the scene		
Excellent (<10 min)	35,339	37.1
Good (10–15 min)	21,159	22.2
Poor (>15 min)	38,874	40.8
Reach hospital duration		
Excellent (<30 min)	14,643	24.4
Good (30–60 min)	31,685	52.7
Poor (>60 min)	13,774	22.9
In-hospital duration		
Excellent (<10 min)	23,172	38.6
Good (10–15 min)	10,396	17.3
Poor (>15 min)	26,513	44.1

**Table 3:** Predictors of SRCA’s response time to road traffic accidents in the KSA.

Variables	Acceptance Duration in Seconds				Movement Duration in Seconds				Reach Site Duration in Minutes			
	Coef.	*p*-Value	95% Conf. Interval		Coef.	*p*-Value	95% Conf. Interval		Coef.	*p*-Value	95% Conf. Interval	
**Region**												
Riyadh	0				0				0			
Hail	−12.8	0.000	−14.1	−11.4	−35.6	0.000	−38.3	−33.0	−1.1	0.000	−1.5	−0.7
Mecca	−5.1	0.000	−5.9	−4.2	1.8	0.027	0.2	3.4	−0.1	0.261	−0.4	0.1
Medina	−4.3	0.000	−5.3	−3.3	−17.9	0.000	−19.8	−15.9	0.7	0.000	0.4	1.0
Western Region	−2.1	0.000	−3.0	−1.3	−7.9	0.000	−9.5	−6.3	0.4	0.003	0.1	0.6
Qassim	−10.5	0.000	−11.6	−9.4	−31.9	0.000	−34.0	−29.8	−2.1	0.000	−2.4	−1.7
Aser	−7.4	0.000	−8.2	−6.5	−11.0	0.000	−12.7	−9.3	−0.3	0.034	−0.5	0.0
Jazzan	−12.8	0.000	−14.1	−11.6	−37.4	0.000	−39.8	−35.1	−1.4	0.000	−1.7	−1.0
Al-Baha	−1.7	0.047	−3.4	0.0	4.7	0.004	1.6	7.9	−0.1	0.730	−0.6	0.4
Najran	−7.8	0.000	−9.5	−6.0	−16.4	0.000	−19.8	−13.0	−1.3	0.000	−1.8	−0.8
Al-Jouf	−9.3	0.000	−11.1	−7.6	−22.8	0.000	−26.0	−19.5	−1.6	0.000	−2.1	−1.1
Tabuk	−8.2	0.000	−9.8	−6.7	−20.8	0.000	−23.7	−17.9	−0.4	0.108	−0.8	0.1
North border	−11.9	0.000	−13.9	−9.8	−32.2	0.000	−36.1	−28.3	−3.1	0.000	−3.7	−2.5
**Nationality**												
Saudi	0				0				0			
Non-Saudi	0.6	0.046	0.0	1.2	2.0	0.000	0.9	3.1	0.8	0.000	0.6	0.9
**Gender**												
Male	0				0				0			
Female	−0.9	0.030	−1.7	−0.1	−1.8	0.024	−3.3	−0.2	−0.1	0.250	−0.4	0.1
**Age**												
0–14 years	0				0				0			
15–24 years	−1.3	0.043	−2.5	0.0	−1.0	0.426	−3.3	1.4	−1.7	0.000	−2.0	−1.3
25–34 years	−1.3	0.036	−2.5	−0.1	0.1	0.911	−2.2	2.5	−1.1	0.000	−1.4	−0.7
35–44 years	−1.2	0.077	−2.5	0.1	0.2	0.905	−2.3	2.6	−0.8	0.000	−1.2	−0.4
45–54 years	−0.6	0.403	−2.1	0.8	0.7	0.633	−2.1	3.5	−0.6	0.003	−1.1	−0.2
55 and above	0.1	0.932	−1.5	1.6	1.6	0.300	−1.4	4.5	−0.9	0.000	−1.3	−0.4
**Accident type**												
Collision	0				0				0			
Runover	−1.7	0.000	−2.7	−0.8	−5.1	0.000	−6.9	−3.3	−3.0	0.000	−3.3	−2.8
Rollover	0.4	0.222	−0.3	1.2	3.3	0.000	1.9	4.7	2.3	0.000	2.1	2.5
**Place of accident**												
Inside City	0				0				0			
Outside City	7.0	0.000	6.5	7.6	22.3	0.000	21.2	23.3	4.9	0.000	4.8	5.1

**Table 4:** Predictors of SRCA’s response time to road traffic accidents in the KSA.

Variables	Onsite Treatment Start Time in Seconds				Duration at Site in Minutes				Reach Hospital Duration in Minutes				In-Hospital Duration			
	Coef.	*p*-Value	95% Conf. Interval		Coef.	*p*-Value	95% Conf. Interval		Coef.	*p*-Value	95% Conf. Interval		Coef.	*p*-Value	95% Conf. Interval	
**Region**																
Riyadh	0				0				0				0			
Hail	−56.0	0.000	−69.6	−42.5	−8.9	0.000	−9.4	−8.3	−12.1	0.000	−13.3	−10.9	−14.0	0.000	−14.7	−13.2
Mecca	9.0	0.032	0.8	17.3	0.0	0.908	−0.3	0.3	−1.3	0.002	−2.1	−0.5	−4.2	0.000	−4.7	−3.7
Medina	−1.4	0.786	−11.6	8.8	−3.4	0.000	−3.8	−3.0	−1.7	0.001	−2.7	−0.7	−1.8	0.000	−2.3	−1.2
Western Region	−9.1	0.032	−17.5	−0.8	−3.8	0.000	−4.1	−3.4	−4.1	0.000	−4.9	−3.2	−9.2	0.000	−9.7	−8.7
Qassim	−26.6	0.000	−37.5	−15.7	−5.0	0.000	−5.4	−4.6	−11.0	0.000	−12.1	−10.0	−11.0	0.000	−11.7	−10.4
Aser	22.4	0.000	13.6	31.2	−1.8	0.000	−2.2	−1.5	−3.7	0.000	−4.6	−2.9	−1.4	0.000	−1.9	−0.9
Jazzan	−1.5	0.808	−13.7	10.7	−5.0	0.000	−5.5	−4.5	−9.4	0.000	−10.5	−8.3	−6.6	0.000	−7.2	−6.0
Al-Baha	−28.0	0.001	−44.6	−11.4	−5.2	0.000	−5.8	−4.5	−7.8	0.000	−9.3	−6.3	−8.9	0.000	−9.8	−8.0
Najran	−18.2	0.045	−36.0	−0.4	−6.6	0.000	−7.3	−5.9	−6.9	0.000	−8.5	−5.3	−7.6	0.000	−8.6	−6.7
Al-Jouf	−55.4	0.000	−72.4	−38.3	−10.6	0.000	−11.3	−10.0	−17.6	0.000	−19.0	−16.1	−13.1	0.000	−14.0	−12.3
Tabuk	−3.3	0.667	−18.5	11.9	−6.2	0.000	−6.8	−5.6	−5.2	0.000	−6.5	−3.8	−7.6	0.000	−8.4	−6.8
North border	−73.5	0.000	−93.8	−53.2	−10.6	0.000	−11.4	−9.8	−19.1	0.000	−20.8	−17.4	−14.0	0.000	−15.0	−12.9
**Nationality**																
Saudi	0				0				0				0			
Non-Saudi	3.2	0.283	−2.6	8.9	0.4	0.000	0.2	0.6	1.8	0.000	1.2	2.3	−0.8	0.000	−1.1	−0.4
**Gender**																
Male	0				0				0				0			
Female	16.6	0.000	8.7	24.5	1.6	0.000	1.3	1.9	3.1	0.000	2.3	3.8	1.1	0.000	0.7	1.6
**Age**																
0–14 years	0				0				0				0			
15–24 years	−7.0	0.264	−19.3	5.3	−0.6	0.018	−1.0	−0.1	−7.6	0.000	−8.8	−6.5	−1.0	0.003	−1.7	−0.4
25–34 years	0.0	0.998	−12.1	12.1	−0.3	0.236	−0.7	0.2	−6.4	0.000	−7.5	−5.2	−1.5	0.000	−2.2	−0.9
35–44 years	−1.3	0.843	−14.2	11.6	0.1	0.824	−0.4	0.6	−5.9	0.000	−7.1	−4.6	−1.3	0.000	−2.0	−0.6
45–54 years	−1.2	0.869	−15.9	13.4	−0.3	0.239	−0.9	0.2	−6.2	0.000	−7.6	−4.9	−1.2	0.003	−2.1	−0.4
55 and above	2.7	0.734	−12.7	18.0	0.4	0.181	−0.2	1.0	−5.1	0.000	−6.6	−3.7	−1.5	0.001	−2.3	−0.6
**Accident type**																
Collision	0				0				0				0			
Runover	−0.9	0.858	−10.3	8.6	−1.5	0.000	−1.8	−1.1	−9.5	0.000	−10.4	−8.7	0.1	0.602	−0.4	0.6
Rollover	18.8	0.000	11.7	26.0	0.7	0.000	0.4	1.0	7.0	0.000	6.3	7.6	0.9	0.000	0.5	1.2
**Place of accident**																
Inside City	0				0				0				0			
Outside city	−28.8	0.000	−34.3	−23.3	−2.2	0.000	−2.4	−2.0	8.6	0.000	8.1	9.1	−3.9	0.000	−4.2	−3.6

## Data Availability

The data set used during the current study is available from the corresponding author upon reasonable request.
